# HoCoRT: host contamination removal tool

**DOI:** 10.1186/s12859-023-05492-w

**Published:** 2023-10-02

**Authors:** Ignas Rumbavicius, Trine B. Rounge, Torbjørn Rognes

**Affiliations:** 1 Centre for Bioinformatics, Department of Informatics, University of Oslo, PO Box 1080 Blindern, 0316 Oslo, Norway; 2 Centre for Bioinformatics, Department of Pharmacy, University of Oslo, PO Box 1068 Blindern, 0316 Oslo, Norway; 3 Cancer Registry of Norway, PO Box 5313 Majorstuen, 0304 Oslo, Norway; 4 Department of Microbiology, Oslo University Hospital, PO Box 4950 Nydalen, 0424 Oslo, Norway

**Keywords:** Microbiome, Shotgun metagenome, Contamination, Classification, Software

## Abstract

**Background:**

Shotgun metagenome sequencing data obtained from a host environment will usually be contaminated with sequences from the host organism. Host sequences should be removed before further analysis to avoid biases, reduce downstream computational load, or ensure privacy in the case of a human host. The tools that we identified, as designed specifically to perform host contamination sequence removal, were either outdated, not maintained, or complicated to use. Consequently, we have developed HoCoRT, a fast and user-friendly tool that implements several methods for optimised host sequence removal. We have evaluated the speed and accuracy of these methods.

**Results:**

HoCoRT is an open-source command-line tool for host contamination removal. It is designed to be easy to install and use, offering a one-step option for genome indexing. HoCoRT employs a variety of well-known mapping, classification, and alignment methods to classify reads. The user can select the underlying classification method and its parameters, allowing adaptation to different scenarios. Based on our investigation of various methods and parameters using synthetic human gut and oral microbiomes, and on assessment of publicly available data, we provide recommendations for typical datasets with short and long reads.

**Conclusions:**

To decontaminate a human gut microbiome with short reads using HoCoRT, we found the optimal combination of speed and accuracy with BioBloom, Bowtie2 in end-to-end mode, and HISAT2. Kraken2 consistently demonstrated the highest speed, albeit with a trade-off in accuracy. The same applies to an oral microbiome, but here Bowtie2 was notably slower than the other tools. For long reads, the detection of human host reads is more difficult. In this case, a combination of Kraken2 and Minimap2 achieved the highest accuracy and detected 59% of human reads. In comparison to the dedicated DeconSeq tool, HoCoRT using Bowtie2 in end-to-end mode proved considerably faster and slightly more accurate. HoCoRT is available as a Bioconda package, and the source code can be accessed at https://github.com/ignasrum/hocort along with the documentation. It is released under the MIT licence and is compatible with Linux and macOS (except for the BioBloom module).

**Supplementary Information:**

The online version contains supplementary material available at 10.1186/s12859-023-05492-w.

## Background

Sequencing the genomes of microbial communities within a host organism's environment has opened new avenues for research into host-microbe interactions. After metagenomic sequencing, several analysis steps are necessary to achieve a comprehensive understanding of the microbiome's composition. Due to the often massive amount of data involved, efficient processing is essential to minimise unnecessary computations. Sequenced data often contains sequences from the host and privacy concerns arise when the host is human. Additionally, non-microbial sequences could introduce bias in downstream analyses. Therefore, the removal of host sequences should be prioritised at an early stage [1].

Decontamination is often managed in an ad-hoc manner by utilising alignment tools to search reads against a host genome database. Ad-hoc approaches may be unnecessarily complicated and could lead to suboptimal performance. The lack of standardised best practices for this procedure hinders comparison across studies.

Some generic metagenome analysis pipelines, such as ATLAS [2] and Sunbeam [3] have integrated modules for host decontamination. ATLAS employs BBsplit [4, 5], while Sunbeam employs BWA [6] as its decontamination method. Using Bowtie2 [7] with the ‘un-conc’ option is also occasionally suggested for host decontamination. With the ‘un-conc’ option, Bowtie2 requires both reads in a pair to map concordantly to the genome.

We could only identify two dedicated tools specifically designed for removing contaminating sequences: DeconSeq and GenCoF. DeconSeq [8] is a command-line tool for identifying and removing sequence contamination from genomic and metagenomic datasets. DeconSeq integrates a modified version of BWA-SW [6], its underlying classifier, directly into its source code, making modifications challenging. DeconSeq supports only single-end Illumina reads, and its code has not been updated since 2013. GenCoF [9] is a graphical user interface for rapidly removing human genome contaminants from metagenomic datasets, limited to short reads. Its GUI nature makes it unsuitable for scripting, and extending GenCoF is difficult as it has Bowtie2 [7] integrated directly into its source code. Both tools have clear limitations, rendering them less suitable for most large-scale datasets. Thus, we developed the new tool HoCoRT to address this gap. To provide recommendations for different circumstances and default settings, we evaluated the performance of various underlying classification methods.

## Implementation

HoCoRT is an open-source command-line-based tool written in Python 3. It is designed to be user-friendly and can be effortlessly installed as a package using Bioconda [10] or as a Docker container. HoCoRT features a modular pipeline design and utilises well-established classification, mapping, and alignment tools to classify sequences into host and non-host (microbial) sequences. The current pipeline modules encompass the BBMap tool in the BBTools suite [4, 5], BioBloom [11], Bowtie2 [7], BWA-MEM2 [12], HISAT2 [13], Kraken2 [14], and Minimap2 [15]. Moreover, modules can pipe data through different tools sequentially. While users can configure pipeline options, recommended defaults are provided. HoCoRT can be extended by creating new modules that utilise other tools. Additionally, HoCoRT offers a comprehensive Python library with an API that can serve as a backend for other tools. HoCoRT supports both reading and writing optionally compressed FASTQ files. The tool also manages the construction of database index files. Built-in help functions and error messages ensure the tool's documentation is readily accessible. HoCoRT relies on Samtools [16]. For a comprehensive list of all software mentioned in this work, including version numbers and references, please refer to Additional file 1: Table S1.

## Evaluation

The classification speed and accuracy of HoCoRT using several different underlying methods and settings were investigated with synthetic and real-world datasets. The GitHub repository at https://github.com/ignasrum/hocort-eval provides the scripts used to generate the synthetic datasets and conduct performance evaluations.

HoCoRT was evaluated on synthetic HiSeq, MiSeq and Nanopore data mimicking human gut and oral microbiomes. The synthetic human gut microbiome datasets comprised a mix of 1% human host sequences and 99% microbial sequences, while the synthetic human oral microbiome datasets included a mix of 50% human host and 50% microbial sequences. Human reads were derived from the Genome Reference Consortium Human Build 38 patch release 13 (GRCh38.p13), while microbial reads were extracted pseudo-randomly from a set of 100 bacterial, fungal, and viral sequences from NCBI GenBank [17]. Accession numbers for the microbial genome sequences are provided in the GitHub repository. To assess the variance in performance, seven different datasets (using distinct random seeds) were generated for each of the six combinations of microbiome (gut and oral) and sequencing technology (HiSeq, MiSeq, and Nanopore), resulting in a total of 42 datasets. Each synthetic short read dataset contains 5 million read pairs randomly generated using InSilicoSeq [18] with the prebuilt HiSeq (2 × 125 bp) and MiSeq (2 × 300 bp) error profiles, while each long read dataset contains 2.5 million single-ended reads generated using NanoSim [19] (average 2159 bp, range 54–98,320 bp). The ‘read profile’ used by NanoSim was generated from the NCBI Sequence Read Archive (SRA) dataset with accession ERR3279199. This dataset consists of unpaired human Nanopore MinION sequencing reads, more specifically, the NA12878 sample and another individual with ataxia-pancytopenia syndrome. The Nanopore basecaller chosen was Guppy.

Seventeen pipelines were examined using Illumina data: Seal, BBDuk, BBSplit, BioBloom, Bowtie2 in end-to-end and local mode, both with and without the ‘un-conc’ option, HISAT2, Kraken2, BBMap in default and fast mode, BWA-MEM2, Kraken2 followed by Bowtie2 in end-to-end mode, Kraken2 followed by HISAT2, Minimap2, and finally Kraken2 followed by Minimap2. Four pipelines were examined using Nanopore data: BioBloom, Minimap2, Kraken2 followed by Minimap2, and Kraken2. CONSULT [20] was considered, but the lack of pre-compiled binaries or packages and its considerable memory requirements make it impractical. CLARK [21] was also considered, but not included due to its primary taxonomic classification focus. The newer Kraken2 tool has been shown to be many times faster and much less memory-demanding than CLARK without any loss of accuracy [14].

The ability to detect human host sequences was tested, and the sensitivity, precision, and accuracy were calculated. True positives (TP) were sequences correctly identified as human, while false positives (FP) were sequences incorrectly identified as human. True negatives (TN) represented sequences correctly identified as microbial, and false negatives (FN) were sequences incorrectly identified as microbial. Sensitivity was calculated as TP/(TP + FN), precision was calculated as TP/(TP + FP), and accuracy was calculated as (TP + TN)/(TP + FP + TN + FN). Given the synthetic human gut microbiome datasets’ 1% human sequences, accuracy here primarily reflects precision, while the accuracy calculated for the oral microbiome datasets is more balanced. When recommending tools, accuracy was prioritised, followed by speed. Performance analysis utilised a Snakemake pipeline [22] on a desktop PC with an AMD Ryzen 7 1700X 8 core/16 thread 3.4 GHz CPU, 64 GB RAM and 4 TB HDD running Linux. No quality filtering or other pre-processing was conducted.

HoCoRT’s performance was compared to DeconSeq using two synthetic human gut datasets with 5 million single-ended short reads each; one with HiSeq and one with MiSeq reads. They were generated as described above, but with single-ended reads, due to the inability of DeconSeq to handle paired-end reads.

The performance of HoCoRT was also evaluated using two real human gut microbiome datasets from the SRA. The first dataset (SRR18498477) consists of gut microbiomes from people living with HIV sequenced using Illumina HiSeq technology. The second dataset (SRR9847864) comprises three healthy human gut microbiome samples sequenced using Oxford Nanopore Technology. We employed BLAST [23] in MegaBLAST mode with an E-value threshold of 1∙10^–10^ and HoCoRT with both Bowtie2 and Minimap2 pipelines to assess the amount of remaining human host contamination.

## Results and discussion

**Fig. 1:**
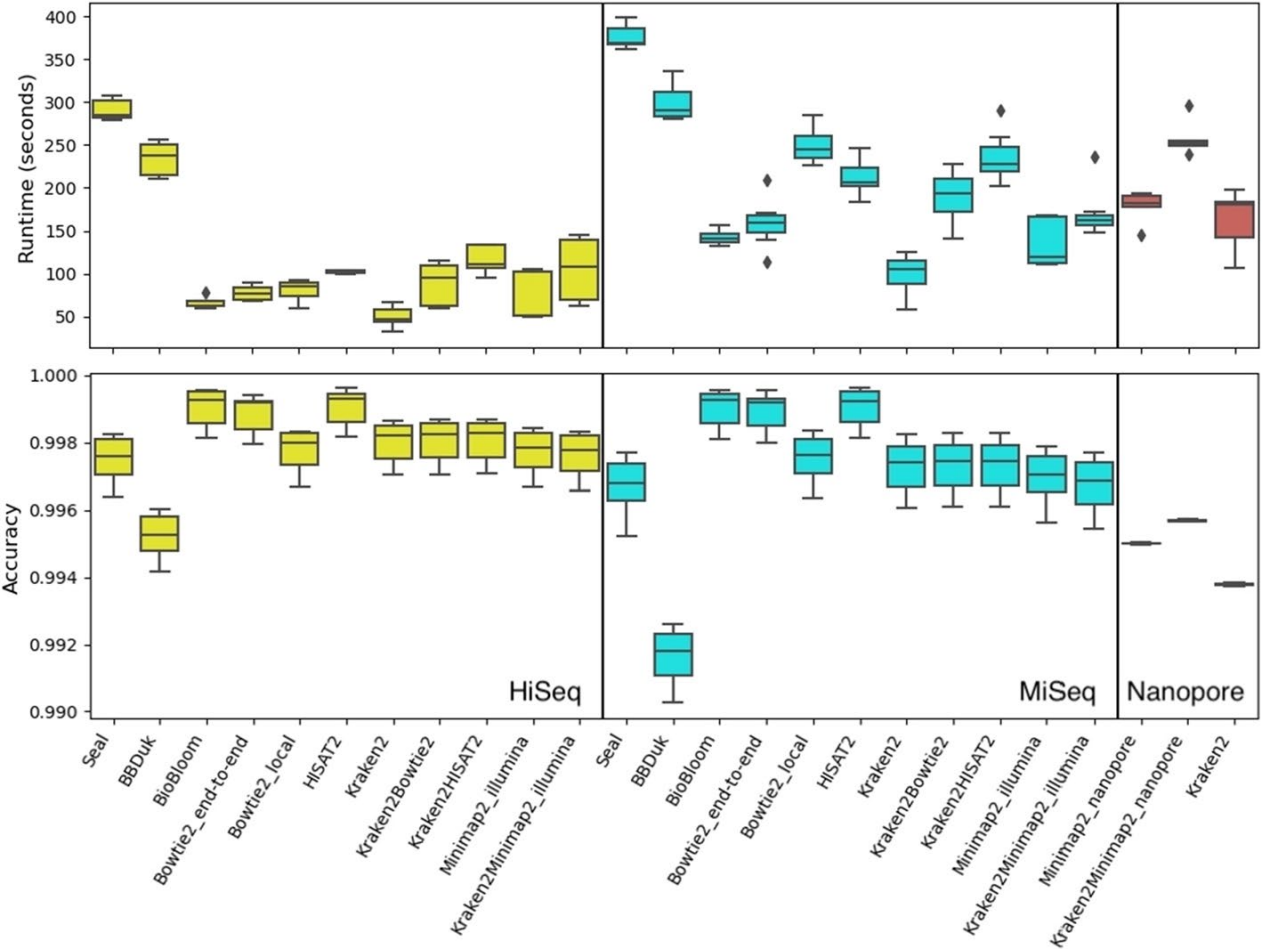
Overview of HoCoRT performance on simulated gut microbiome datasets. Box plots of HoCoRT runtime in seconds (top) and classification accuracy (bottom) using several different classification modules and parameters on Illumina HiSeq (yellow, left), MiSeq (cyan, middle) and Nanopore data (red, right). Table 1 contains additional results, including those for BioBloom (on Nanopore data), BBMap, BBSplit, Bowtie2 with the ‘un-conc’ option, and BWA-MEM2, which were excluded from this figure due to outliers

The classification speed and accuracy of HoCoRT on the synthetic gut microbiome are shown in Fig. 1 and Table 1. Overall, BioBloom, Bowtie2 in end-to-end mode, and HISAT2 performed best for short reads and are recommended due to high accuracy and speed across scenarios. The best tools detect almost all human short reads, but also incorrectly include a small number of bacterial reads. Kraken2 consistently displayed the highest speed with a minor reduction in accuracy. For long reads, the sensitivity decreased substantially, with only 59% of human reads detected in the best-case scenario, achieved by a combination of Kraken2 and Minimap2. Synthetic oral microbiome results are presented in Additional file 1: Fig. S1 and Table S2. These results are similar to the gut microbiome results, but Bowtie2 was clearly slower than the other options, while also the most accurate, in particular for the MiSeq reads. This may be due to the higher number of aligned human sequences.

**Table 1:** Detailed HoCoRT performance on simulated human gut microbiome datasets

Pipeline	Runtime	Accuracy	Precision	Sensitivity
Paired-end HiSeq				
Seal	291.3	0.9975	0.8027	**1.0000**
BBDuk	233.7	0.9952	0.6786	**1.0000**
BBSplit	509.0	0.9982	0.8523	**1.0000**
BioBloom	66.6	**0.9990**	0.9143	0.9995
Bowtie2_end-to-end	77.4	0.9988	0.8978	**1.0000**
Bowtie2_local	80.4	0.9978	0.8187	**1.0000**
Bowtie2_end-to-end_un_conc	277.2	0.9934	**0.9351**	*0.3625*
Bowtie2_local_un_conc	314.9	0.9941	0.8956	0.4614
HISAT2	101.7	**0.9990**	0.9145	0.9998
Kraken2	**49.8**	0.9980	0.8385	0.9928
BBMap_default	*1053.2*	0.9982	0.8520	**1.0000**
BBMap_fast	300.9	0.9986	0.8762	0.9999
BWA_MEM2	381.3	*0.9720*	*0.2635*	**1.0000**
Kraken2Bowtie2	87.7	0.9980	0.8385	**1.0000**
Kraken2HISAT2	117.2	0.9980	0.8388	**1.0000**
Minimap2_illumina	73.3	0.9977	0.8170	**1.0000**
Kraken2Minimap2_illumina	105.2	0.9976	0.8107	**1.0000**
Paired-end MiSeq				
Seal	376.7	0.9967	0.7559	**1.0000**
BBDuk	299.7	0.9916	0.5457	**1.0000**
BBSplit	791.9	0.9985	0.8726	**1.0000**
BioBloom	142.0	**0.9990**	0.9129	0.9969
Bowtie2_end-to-end	159.0	0.9989	0.9041	0.9999
Bowtie2_local	249.8	0.9975	0.8043	**1.0000**
Bowtie2_end-to-end_un_conc	747.3	0.9904	**0.9721**	*0.0457*
Bowtie2_local_un_conc	810.6	0.9919	0.8761	0.2243
HISAT2	212.6	**0.9990**	0.9224	0.9901
Kraken2	**99.0**	0.9973	0.7902	0.9960
BBMap_default	2338.7	0.9985	0.8730	0.9993
BBMap_fast	733.3	0.9989	0.9044	0.9956
BWA_MEM2	*2889.4*	*0.9128*	*0.1032*	**1.0000**
Kraken2Bowtie2	189.2	0.9973	0.7908	**1.0000**
Kraken2HISAT2	236.2	0.9973	0.7908	**1.0000**
Minimap2_illumina	136.5	0.9970	0.7698	**1.0000**
Kraken2Minimap2_illumina	170.9	0.9967	0.7567	**1.0000**
Single-end Nanopore				
BioBloom	171.6	*0.9900*	**1.0000**	*0.0013*
Minimap2_nanopore	179.7	0.9950	0.9965	0.5027
Kraken2Minimap2_nanopore	*256.3*	**0.9957**	0.9632	**0.5916**
Kraken2	**162.4**	0.9938	*0.9491*	0.3994

The average runtime (in seconds), accuracy, precision, and sensitivity are shown for each pipeline and for each data type. The best (bold) and worst (italic) performing pipelines are indicated for each performance metric and data type

HoCoRT’s performance was compared to DeconSeq using human gut datasets with short reads. The HoCoRT Bowtie2 (end-to-end) pipeline exhibited substantially higher alignment speed than DeconSeq for both HiSeq (34X) and MiSeq (49X) reads, and slightly better accuracy, as shown in Additional file 1: Table S3.

Lastly, HoCoRT’s performance was evaluated using two real human gut microbiome datasets. Up to 0.03% of the reads in these datasets were identified as human, as shown in Additional file 1: Table S4. Minimap2 identified the highest number of reads, followed by BLAST and then Bowtie2. BLAST required about 40 times more time than the other tools. Since the true number of human reads is unknown, we cannot calculate sensitivity and precision. Based on the results from the synthetic datasets, most of the true human reads are probably detected, but how many of the predicted human reads that really are microbial is difficult to estimate.

For short reads, based on the overall very high sensitivity of most tools, it appears that almost all human host sequences can be detected reliably in microbiomes, while a small number of microbial sequences may be incorrectly classified as human. If necessary, some precision may be traded-off for decreased run-time, depending on the specific use case and how important it is to keep as many microbial sequences as possible.

For long reads, the situation is more challenging and only about 59% of the actual human host reads were detected in the best-case scenario. Improved tools are required to reliably detect human host contamination in long reads with the error profiles studied.

Additional results and a comprehensive description of HoCoRT can be found in the first author’s master’s thesis [24].

## Conclusions

A dedicated, flexible, extendable, and modular tool for removing host sequence contamination is now available, free of charge. We have conducted a comprehensive comparison of classification methods and offer corresponding recommendations. The HoCoRT tool is expected to streamline the decontamination step in microbiome data analysis and deliver reliable performance.

### Availability and requirements

Project name: HoCoRT. Project home page: https://github.com/ignasrum/hocort. Operating system(s): Linux and macOS (except for the BioBloom module). Programming language: Python. Other requirements: Samtools [14] and other packages. Please see GitHub repository for details. License: MIT license. Any restrictions to use by non-academics: None.

### Supplementary Information

**Additional file 1:** Supplementary figure and tables.

## Data Availability

The scripts used for evaluation are available at https://github.com/ignasrum/hocort-eval and include a list of the accession numbers of the bacterial genome sequences included in the synthetic microbiomes. The two real human gut microbiomes are available at the Sequence Read Archive with accession numbers SRR18498477 and SRR9847864.
