# Supplementary material for: HoCoRT: host contamination removal tool
Source: BMC Bioinformatics. 2023 Oct 2;24:371. doi: 10.1186/s12859-023-05492-w (PMC10544359; doi:10.1186/s12859-023-05492-w)
Supplement: Supplementary file 1 — Additional file 1: Supplementary figure and tables. [file 12859_2023_5492_MOESM1_ESM.pdf]

HoCoRT: Host contamination removal tool

Ignas Rumbavicius, Trine B. Rounge, Torbjørn Rognes

Supplementary material

Supplementary figures

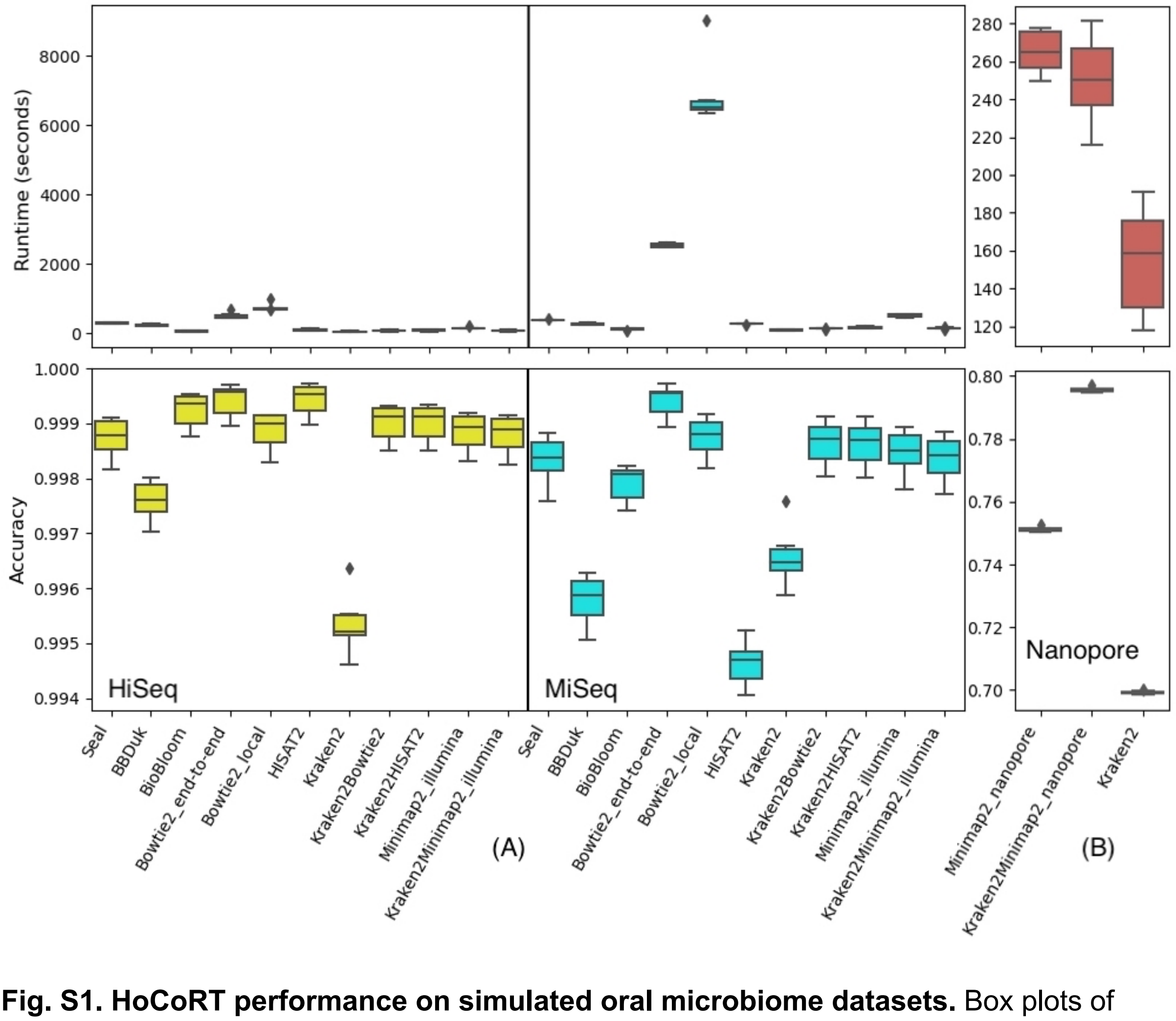

Fig. S1. HoCoRT performance on simulated oral microbiome datasets. Box plots of HoCoRT runtime in seconds (top) and classification accuracy (bottom) using several different classification modules and parameters on (A) HiSeq (yellow, left), MiSeq (cyan, right) and (B) Nanopore data (red). Table S2 contains additional results, including those for BioBloom (on Nanopore data), BBMap, BBSplit, Bowtie2 with the “un-conc” option, and BWA-MEM2, which were excluded from this figure due to outliers.

Supplementary tables

Table S1. Software. The software packages used or mentioned are listed with version numbers and references.

Software	Version	References
ATLAS		[2]
BBDuk	39.01	[4, 5]
BBMap	39.01	[4, 5]
BBSplit	39.01	[4, 5]
BioBloom Tools	2.3.5	[11]
BioConda	23.1.0	[10]
BLAST	2.13.0	[23]
Bowtie2	2.4.5	[7]
BWA-SW		[6]
BWA-MEM2	2.2.1	[12]
CLARK		[21]
CONSULT		[20]
DeconSeq	0.4.3	[8]
GenCoF		[9]
HISAT2	2.2.1	[13]
HoCoRT	1.2.2	[24]
InSiliconSeq	1.5.4	[18]
Kraken2	2.1.2	[14]
MiniMap2	2.24	[15]
NanoSim	3.0.0	[19]
Samtools	1.16.1	[16]
Seal	39.01	[4, 5]
SnakeMake	7.19.1	[22]
Sunbeam		[3]

Table S2. Detailed HoCoRT performance on simulated oral microbiome datasets. The average runtime (in seconds), accuracy, precision, and sensitivity of the classification are shown for each pipeline and for each data type. The best (blue) and worst (red) performing pipelines are indicated for each performance metric and data type.

Pipeline	Runtime	Accuracy	Precision	Sensitivity
**Paired-end HiSeq**				
Seal	289.5	0.9987	0.9975	1.0000
BBDuk	228.2	0.9976	0.9952	1.0000
BBSplit	1489.1	0.9991	0.9982	1.0000
BioBloom	60.0	0.9992	0.9990	0.9994
Bowtie2_end-to-end	495.4	0.9994	0.9988	1.0000
Bowtie2_local	739.4	0.9989	0.9977	1.0000
Bowtie2_end-to-end_un_conc	571.3	0.6809	0.9993	0.3621
Bowtie2_local_un_conc	814.7	0.7300	0.9988	0.4606
HISAT2	104.4	0.9994	0.9990	0.9998
Kraken2	47.5	0.9954	0.9980	0.9927
BBMap_default	9351.0	0.9991	0.9982	1.0000
BBMap_fast	1062.5	0.9992	0.9985	0.9999
BWA_MEM2	461.9	0.9858	0.9725	1.0000
Kraken2Bowtie2	68.1	0.9990	0.9980	1.0000
Kraken2HISAT2	79.9	0.9990	0.9980	1.0000
Minimap2_illumina	149.1	0.9988	0.9977	1.0000
Kraken2Minimap2_illumina	70.9	0.9988	0.9976	1.0000
**Paired-end MiSeq**				
Seal	374.8	0.9983	0.9967	1.0000
BBDuk	273.0	0.9958	0.9916	1.0000
BBSplit	4046.9	0.9993	0.9985	1.0000
BioBloom	116.9	0.9979	0.9990	0.9968
Bowtie2_end-to-end	2540.8	0.9994	0.9989	0.9999
Bowtie2_local	6887.7	0.9988	0.9975	1.0000
Bowtie2_end-to-end_un_conc	2534.2	0.5230	0.9997	0.0460
Bowtie2_local_un_conc	7015.4	0.6122	0.9986	0.2247
HISAT2	267.9	0.9946	0.9991	0.9901
Kraken2	89.4	0.9966	0.9973	0.9959
BBMap_default	84978.6	0.9989	0.9985	0.9992
BBMap_fast	3435.6	0.9973	0.9989	0.9957
BWA_MEM2	2142.9	0.9718	0.9471	1.0000
Kraken2Bowtie2	142.3	0.9986	0.9973	1.0000
Kraken2HISAT2	163.6	0.9986	0.9973	0.9999
Minimap2_illumina	518.5	0.9985	0.9970	1.0000
Kraken2Minimap2_illumina	149.1	0.9984	0.9967	1.0000
**Single-end Nanopore**				
BioBloom	152.1	0.5007	1.0000	0.0013
Minimap2_nanopore	265.3	0.7512	1.0000	0.5025
KrakenMinimap2_nanopore	250.7	0.7957	0.9996	0.5916
Kraken2	153.9	0.6993	0.9995	0.3988

Table S3. Comparison of the performance of DeconSeq and HoCoRT using Bowtie2 in end-to-end mode on single-ended HiSeq and MiSeq reads. The average runtime (in seconds), accuracy, precision, and sensitivity are shown for each tool. The best (blue) and worst (red) performing tool is indicated for each performance metric.

Tool and pipeline	Runtime	Accuracy	Precision	Sensitivity
**Single-end HiSeq**				
Bowtie2_end-to-end	49.8	0.99947	0.94934	0.99992
DeconSeq	1677.2	0.99869	0.88432	0.99994
**Single-end MiSeq**				
Bowtie2_end-to-end	88.7	0.99960	0.96194	0.99974
DeconSeq	4384.2	0.99824	0.85028	1.00000

Table S4. Evaluation on real microbiome datasets. The number of potentially contaminating human reads identified in two real microbiome datasets sequenced using Illumina HiSeq (SRR18498477) and Nanopore (SRR9847864) technology are shown. The HoCoRT pipelines using Bowtie2 and Minimap2 were employed, as well as BLAST. The fraction of predicted human reads are indicated in parentheses as percentages of all the original reads.

**Accession**	SRR18498477	SRR9847864
**Sequencing**	Illumina HiSeq	Nanopore
**Original reads**	46 273 568	9 741 166
**HoCoRT Bowtie2** (of original)	9 704 (0.021%)	-
**HoCoRT Minimap2** (of original)	12 252 (0.026%)	3 288 (0.033%)
**BLAST** ($E < 1 \cdot 10^{-10}$) (of original)	10 970 (0.024%)	154 (0.0016%)
